# Cardiometabolic disorders affect myocardial [^18^F]fluorodeoxyglucose uptake– impact on diagnostic PET/CT imaging

**DOI:** 10.1186/s13550-025-01278-8

**Published:** 2025-07-01

**Authors:** Suvi Hartikainen, Ville Vepsäläinen, Tuomo Tompuri, Tiina M. Laitinen, Marja Hedman, Tomi Laitinen

**Affiliations:** 1 Department of Clinical Physiology and Nuclear Medicine, Kuopio University Hospital, Kuopio, Finland; 2 Institute of Clinical Medicine, University of Eastern Finland, Kuopio, Finland; 3 Heart Centre, Kuopio University Hospital, Kuopio, Finland; 4 Department of Clinical Physiology, North Karelia Central Hospital, Joensuu, Finland

**Keywords:** Beta-hydroxybutyrate, BHB, Myocardium, Insulin resistance, FDG, PET/CT

## Abstract

**Background:**

Corresponding to impaired insulin response in skeletal muscle, insulin resistance may occur in the myocardium, suggesting a link between cardiometabolic disorders and cardiac glucose metabolism. We aimed to investigate whether cardiometabolic disorders predict myocardial [^18^F]fluorodeoxyglucose ([^18^F]FDG) uptake suppression in cardiac positron emission tomography / computed tomography (PET/CT) following a ketogenic diet and fasting.

**Results:**

The study included 100 patients undergoing [^18^F]FDG-PET/CT following a ketogenic diet of 1–2 days and a 12 h fast. Blood glucose, insulin, β-hydroxybutyrate (BHB), cholesterol, triglycerides and free fatty acid (FFA) levels were measured before [^18^F]FDG injection and later off-diet following overnight fast. The homeostatic model assessment–insulin resistance (HOMA-IR) value was calculated. Non-contrast computed tomography was used to assess the presence of fatty liver and visceral and subcutaneous fat areas. Suppression of myocardial [^18^F]FDG uptake was considered adequate if myocardial uptake was equal to or lower than the blood pool background. Compared to inadequate suppression, adequate suppression was associated with higher levels of BHB (median 0.26 mmol/l for inadequate and 0.43 mmol/l for adequate suppression, *p* = 0.004), FFA (0.58 mmol/l and 0.76 mmol/l respectively, *p* < 0.001), triglycerides (0.87 mmol/l and 1.12 mmol/l respectively, *p* = 0.028), and lower liver-spleen attenuation ratios (1.17 and 1.04 respectively, *p* = 0.013). Low HDL cholesterol, high triglycerides and off-diet HOMA-IR, as well as visceral adiposity, fatty liver and hypertension, predicted adequate suppression in men. Elevated BHB and FFA were significant predictors of adequate suppression in women.

**Conclusions:**

Cardiometabolic disorders are associated with lower myocardial [^18^F]FDG uptake. Insulin resistance and several other cardiometabolic risk factors are associated with attenuated uptake, especially in men. In women, factors reflecting metabolic response to a ketogenic diet and fasting have a more pronounced effect on myocardial [^18^F]FDG uptake.

## Background

Type 2 diabetes mellitus (T2DM) is known to alter the biodistribution of [^18^F]fluorodeoxyglucose ([^18^F]FDG), likely because of an excessive amount of non-radioactive blood glucose as well as insulin resistance, in patients with T2DM [1–3]. However, metabolic disturbances other than T2DM can alter glucose metabolism in muscles, including the myocardium. One notable example of this type of metabolic disturbance is fatty liver, which is associated with lower [^18^F]FDG uptake in the myocardium [4–6]. Similarly, visceral adiposity, a hallmark of metabolic syndrome, is also reportedly associated with lower than normal myocardial [^18^F]FDG uptake [7]. Multiple metabolic syndrome traits have recently been linked with lower myocardial uptake, while an improved metabolic profile has been associated with normalisation of myocardial [^18^F]FDG uptake [8]. However, these metabolic syndrome studies have not been performed in a high-fat, low-carbohydrate (i.e. a ketogenic) diet setting.

This lack of data on the effects of cardiometabolic syndrome during a ketogenic diet has particular relevance to the diagnosis and monitoring of inflammatory cardiac diseases using [^18^F]FDG positron emission tomography ([^18^F]FDG-PET/CT), where the physiological myocardial uptake of the injected [^18^F]FDG must be suppressed. To achieve this suppression, the current guidelines recommend that, prior to the [^18^F]FDG injection, patients consume a high-fat diet lacking carbohydrates (i.e. essentially a ketogenic diet) for 12–24 h, followed by fasting for 12–18 h, with or without intravenous unfractionated heparin (50 IU/kg) 15 min prior to the injection [9, 10]. Even with these dietary precautions, suppression may still be inadequate and may hamper the diagnosis or exclusion of inflammatory cardiac diseases [11, 12].

The aim of our study was to evaluate how consistently the determination of impaired myocardial glucose uptake reflects the presence of an underlying cardiometabolic disorder. A second aim was to validate our previously published pretest calculator of inadequate myocardial suppression [13]. The results provide insight into the regulation of myocardial metabolism and may contribute to the development of the preparation protocol for cardiac [^18^F]FDG PET/CT.

## Methods

### Patient preparation

Between December 2022 and June 2024, 100 adult patients with a clinical indication for cardiac [^18^F]FDG PET/CT were recruited. Patients were not recruited if intravenous unfractionated heparin could not be used (i.e. the patients were receiving ongoing anticoagulation therapy or had a significant risk for bleeding. The study was performed in compliance with the Declaration of Helsinki and was approved by the local ethics committee. Written informed consent was obtained from all subjects.

All patients had been on a low-carbohydrate, high-fat diet for 1–2 days and had fasted for at least 12 h before [^18^F]FDG injection. Their food diaries had also been checked before recruitment. Their blood glucose levels were measured using Accu-Chek Performa (Roche Diagnostics GmbH, Mannheim, Germany) glucose meters, and their blood β-hydroxybutyrate (BHB) levels were determined using Freestyle Precision NEO (Abbott Diabetes Care Ltd., Witney, Oxon, UK) point-of-care devices. Blood was drawn for determinations of glucose, insulin, BHB, total cholesterol, high- and low-density lipoprotein cholesterol (HDL-C and LDL-C) and triglyceride levels and for the analysis of free fatty acids (FFA).

If the point-of-care BHB level was less than 0.35 mmol/l [13], the patients received unfractionated heparin (50 IU/kg) intravenously 15 min before [^18^F]FDG injection. PET/CT imaging started from the thighs or ankles 60 min after [^18^F]FDG injection.

### Laboratory assays

Insulin levels were analysed using an electrochemiluminescence immunoassay, glucose levels with an enzymatic method, FFAs with a photometric method, and total cholesterol, HDL-C, LDL-C, and triglycerides using photometric enzymatic methods. Homeostatic model– insulin resistance (HOMA-IR) was calculated using the following formula, with glucose levels in mmol/l and insulin levels in mU/l [14]:

$$\:HOMA-IR=\frac{glucose\times\:insulin}{22.5}$$

For our analysis of off-diet fasting glucose, insulin, BHB, total cholesterol, HDL-C, LDL-C, and triglyceride levels, the patients were asked to undergo a second, 12 h fasting blood draw about two weeks after returning to their regular diet. HOMA-IR was calculated from the samples using the formula shown above.

### Data acquisition

All patients were scanned using the Biograph Vision PET/CT (Siemens Healthineers, Knoxville, TN, USA) system. Images were reconstructed using iterative reconstruction with four iterations and time-of-flight and point spread function corrections.

Hybrid Viewer (Hermes Medical Solutions, Stockholm, Sweden) was used to analyse the PET images. Images were tilted according to the cardiac planes for cardiac [^18^F]FDG uptake measurements. Four circular regions of interest (ROIs), 1 cm in diameter, were drawn in the ventricular septum, along with four in the lateral wall and one in the left chamber blood pool, using the four-chamber view. In the cases with inflammatory cardiac diseases, focal uptake areas were excluded from the analysis. Myocardial uptake was measured as the maximum standardised uptake value (SUVmax). The mean of the SUVmax values of the left ventricle myocardial ROIs was calculated (SUV_LV_). The ratio of the SUV_LV_ to the left ventricle blood pool (SUVratio) was calculated, and an SUVratio ≤ 1 was considered to indicate adequate myocardial suppression.

Liver and spleen measurements were made from axial CT stacks using the Hybrid Viewer. Circular ROIs, 2 cm in diameter, were drawn in the posterior right lobe, anterior right lobe, and left lobe of the liver, and one 2 cm ROI was drawn in the spleen. Liver attenuation was calculated as the mean Hounsfield units (HU) of the anterior and posterior right lobe ROIs. Fatty liver was defined as a liver attenuation to spleen attenuation ratio (liver–spleen attenuation ratio) of less than 1 [15, 16].

Subcutaneous and visceral fat areas were measured from a 3-mm CT slice at the L3–L4 disc level [17] using Syngo.via (Siemens Healthineers). The Anatomy visualizer Segmentator was used to draw ROIs following the contours of the skin and muscle fascia. The subcutaneous layer was defined as the region between the skin and the fascia, while the visceral fat layer was defined as the region internal to the fascia. An automatic threshold of -200 to -40 HU was applied to identify adipose tissue.

The electronic patient record system was searched for inflammatory cardiac disease, hypertension, dyslipidaemia, diabetes and the use of sodium-glucose cotransporter 2 (SGLT2) inhibitors.

### Statistical analysis

Statistical analysis was performed using the IBM SPSS Statistics (version 29.0.1.0, IBM Corp., Armonk, NY, USA). Because many variables were not normally distributed even after logarithmic correction, continuous variables are represented as median values with minimum and maximum values, and categorical variables as counts with percentages. Spearman’s ρ was used to evaluate bivariate correlations between the SUVratio and other variables. Continuous variables were tested using the Mann–Whitney U test, and categorical variables were tested using the Pearson χ^2^ test. A subgroup analysis was performed among women and men.

The pretest probability calculator of inadequate suppression has been published previously [13]. The factors in the calculator are obesity defined by body mass index (BMI, in kg per square of height in m) ≥ 30, BHB level, T2DM, use of SGLT2 inhibitors and a ketogenic diet of at least one day.

The ability of the point-of-care BHB test and the pretest calculator to predict myocardial glucose metabolism suppression below the left ventricle blood pool was evaluated by receiver operating characteristic (ROC) curve analysis. A *p* value of 0.05 was considered statistically significant. The DeLong test was used to test the difference between the AUC values of the BHB test and the pretest probability calculator.

## Results

### Patient characteristics

The clinical characteristics of the patients are shown in Table 1. The median (min–max) age of the study population was 58 (23–79) years. In the study population (*n* = 100), 53 (53%) were male. The on-diet median blood BHB level obtained using the point-of-care device (0.4 [0.1–5.8] mmol/l) was comparable to the level obtained with the laboratory assay (0.37 [< 0.01–2.49] mmol/l) (*r* = 0.789, *p* < 0.001). BHB and FFA values showed a positive correlation (*r* = 0.557, *p* < 0.001).

**Table 1:** Clinical characteristics of the overall study population and the sex-specific subgroups (men and women)

	Whole study population	male (*n* = 53)	female (*n* = 47)	*p*
Age (years)	58 (23–79)	58 (23–79)	56 (40–75)	0.774
**On-diet laboratory assays**				
POC BHB (mmol/l)	0.4 (0.1–5.8)	0.3 (0.1–5.8)	0.4 (0.1-3.0)	0.068
BHB (mmol/l)^a^	0.37 (0.00-2.49)	0.30 (< 0.01–2.11)	0.41 (< 0.01–2.49)	0.351
FFA (mmol/l)^b^	0.72 (0.00-1.44)	0.64 (0.33–1.40)	0.81 (< 0.01–1.44)	**0.010**
Glucose (mmol/l)^c^	5.1 (3.7–10.2)	5.1 (3.7–10.2)	5.0 (4.0-7.1)	0.055
Insulin (mU/l)^c^	7.9 (1.5–56.6)	8.4 (1.5–56.6)	7.0 (1.5–41.9)	0.248
Total cholesterol (mmol/l)^c^	4.3 (2.1–6.8)	4.1 (2.1–6.8)	4.6 (2.1–6.5)	0.257
HDL-C (mmol/l)^c^	1.40 (0.47–3.25)	1.22 (0.47–2.18)	1.61 (0.67–3.25)	**< 0.001**
LDL-C (mmol/l)^c^	2.6 (0.4–4.9)	2.7 (0.7–4.9)	2.6 (0.4–4.8)	0.905
Triglycerides (mmol/l)^c^	0.99 (0.34–7.33)	1.08 (0.48–7.33)	0.86 (0.34–3.54)	0.151
HOMA-IR^c^	1.77 (0.28–13.22)	1.93 (0.28–12.83)	1.51 (0.28–13.22)	0.167
**Off-diet laboratory assays** ^d^				
BHB (mmol/l)	< 0.01 (< 0.01–0.54)	< 0.01 (< 0.01–0.46)	< 0.01 (< 0.01–0.54)	0.201
Glucose (mmol/l)	5.6 (4.6–7.7)	5.6 (4.9–7.7)	5.6 (4.6–7.3)	0.124
Insulin (mU/l)	11.1 (2.5–88.8)	13.5 (3.6–88.8)	10.3 (2.5–38.9)	0.264
Total cholesterol (mmol/l)	4.7 (2.4–7.4)	4.6 (3.0-7.4)	5.1 (2.4–7.4)	0.449
HDL-C (mmol/l)	1.47 (0.63–3.09)	1.26 (0.63–1.79)	1.65 (0.83–3.09)	**0.002**
LDL-C (mmol/l)	2.8 (0.5–5.3)	2.9 (1.6–5.3)	2.8 (0.5–5.2)	0.589
Triglyceride (mmol/l)	1.38 (0.5–3.73)	1.38 (0.52–3.73)	1.38 (0.50–3.60)	0.880
HOMA-IR	2.54 (0.63–23.68)	3.72 (0.85–23.68)	2.37 (0.63–12.62)	0.179
**Body composition**				
BMI (kg/m^2^)	29.1 (18.4–54.4)	28.9 (19.4–54.4)	29.4 (18.4–47.9)	0.450
Visceral fat area (cm^2^)	15.41 (1.56–54.89)	19.99 (4.51–52.11)	12.69 (1.56–54.89)	**0.007**
Subcutaneous fat area (cm^2^)	27.12 (5.16–81.98)	22.14 (5.74–59.30)	32.79 (5.16–81.98)	**0.005**
Liver attenuation (HU)	47.6 (-8.5-63.8)	44.87 (3.04–63.78)	49.57 (-8.49-58.86)	0.062
Liver-to-spleen ratio	1.08 (-0.19-2.75)	1.05 (0.07–2.75)	1.13 (-0.19-1.81)	0.215
**Cardiometabolic risk factors**				
Type 2 diabetes	9 (9%)	4 (8%)	5 (11%)	0.590
Obesity*	41 (41%)	20 (38%)	21 (45%)	0.481
Hypertension	35 (35%)	20 (38%)	15 (32%)	0.542
Dyslipidaemia	45 (45%)	24 (45%)	21 (45%)	0.952
Fatty liver	36 (36%)	22 (42%)	14 (30%)	0.223

Values are presented as medians (range) for continuous variables and as counts (percentages) for discreate variables. *p* < 0.05 is considered significant (bolded). *POC*, a point-of-care device; *BHB*, beta-hydroxybutyrate; *FFA*, free fatty acids; *HDL-C*, high-density lipoprotein cholesterol; *LDL-C*, low-density lipoprotein cholesterol; *HOMA-IR*, homeostatic model assessment– insulin resistance; *BMI*, body mass index; *HU*, Hounsfield units. *Using WHO classification, BMI ≥ 30. a, *n* = 85; b, *n* = 95; c, *n* = 99; d, *n* = 51; all others, *n* = 100

Fifty-one patients underwent repeat laboratory tests 15 (4–239) days after imaging, following an overnight fast.

The median values for patient BMI, visceral fat area and subcutaneous fat area were 29.1 (18.4–54.4), 15.4 (1.6–54.9) cm^2^, and 27.1 (5.2–82.0) cm^2^, respectively. The median liver attenuation was 47.55 (− 8.49 to 63.78) HU, and the median liver–spleen ratio was 1.08 (− 0.19 to 2.75). Overall, 36 patients (36%) had fatty liver.

Forty-one patients (41%) were obese, 35 (35%) had hypertension, 45 (45%) had been diagnosed with dyslipidaemia and prescribed statins, nine (9%) had T2DM, 21 (21%) used SGLT2 inhibitors, and 16 (16%) were on systemic corticosteroids. Of the patients with T2DM, five were on metformin and five used a gliptin, a glutide, or a combination of those. Of those without T2DM, one used a glutide for obesity indication.

Associations between metabolic risk factors and the myocardial uptake ratio.

The median SUVratio was 0.80 (0.41–12.94). The correlations detected between the SUVratio and different variables are listed in Table 2. Statistically significant correlations were observed between the SUVratio and the levels of BHB, FFA, glucose, LDL-C and triglycerides, and between the SUVratio and liver attenuation, and the liver–spleen attenuation ratio. The SUVratio showed an inverse correlation with the visceral fat area, but only in men, whereas it showed a positive correlation only with the BHB and FFA levels in women.

**Table 2:** Correlations between the SUV ratio and different variables

	Whole study population		Men		Women	
	coefficient	*p*	coefficient	*p*	coefficient	*p*
Age (years)	-0.007	0.945	-0.043	0.760	0.038	0.798
**On-diet laboratory assays**						
POC BHB (mmol/l)	-0.282	**0.005**	-0.290	**0.035**	-0.321	**0.028**
BHB (mmol/l)^a^	-0.343	**0.001**	-0.299	**0.046**	-0.447	**0.004**
FFA (mmol/l)^b^	-0.378	**< 0.001**	-0.430	**0.002**	-0.422	**0.004**
Glucose (mmol/l)^c^	0.214	**0.033**	0.243	0.080	0.247	0.098
Insulin (mU/l)^c^	-0.153	0.131	-0.315	**0.021**	0.072	0.636
Total cholesterol (mmol/l)^c^	0.164	0.104	0.221	0.080	0.111	0.462
HDL-C (mmol/l)^c^	0.143	0.158	0.243	0.079	0.005	0.971
LDL-C (mmol/l)^c^	0.214	**0.034**	0.271	**0.050**	0.184	0.222
Triglycerides (mmol/l)^c^	-0.302	**0.002**	-0.339	**0.013**	-0.281	0.058
HOMA-IR^c^	-0.128	0.208	-0.274	**0.047**	0.101	0.503
**Off-diet laboratory assays** ^d^						
BHB (mmol/l)	-0.251	0.108	-0.413	0.088	-0.032	0.883
Glucose (mmol/l)	0.014	0.921	0.200	0.359	-0.148	0.453
Insulin (mU/l)	-0.331	**0.017**	-0.289	0.182	-0.245	0.209
Total cholesterol (mmol/l)	0.254	0.072	0.337	0.115	0.184	0.348
HDL-C (mmol/l)	0.104	0.466	0.129	0.559	-0.020	0.919
LDL-C (mmol/l)	0.323	**0.021**	0.362	0.090	0.314	0.104
Triglyceride (mmol/l)	-0.025	0.860	0.140	0.523	-0.135	0.493
HOMA-IR	-0.330	**0.018**	-0.323	0.133	-0.252	0.195
**Body composition**						
BMI	-0.019	0.855	-0.262	0.058	0.217	0.143
CT visceral fat area (cm^2^)	-0.170	0.090	-0.359	**0.008**	0.135	0.367
CT subcutaneous fat area (cm^2^)	-0.004	0.970	-0.243	0.079	0.202	0.174
Liver attenuation (HU)	0.267	**0.007**	0.436	**0.001**	0.012	0.937
Liver-to-spleen ratio	0.245	**0.014**	0.300	**0.029**	0.171	0.249

*p* < 0.05 is considered significant (bolded). SUVratio, ratio of standardized uptake values in myocardium and left ventricular blood pool; *POC*, a point-of-care device; *BHB*, beta-hydroxybutyrate; *FFA*, free fatty acids; *HDL-C*, high-density lipoprotein cholesterol; *LDL-C*, low-density lipoprotein cholesterol; *HOMA-IR*, homeostatic model assessment– insulin resistance; *BMI*, body mass index; *HU*, Hounsfield units. a, *n* = 85; b, *n* = 95; c, *n* = 99; d, *n* = 51; all others, *n* = 100

### Factors associated with the state of myocardial [^18^F]FDG suppression

**Fig. 1:**
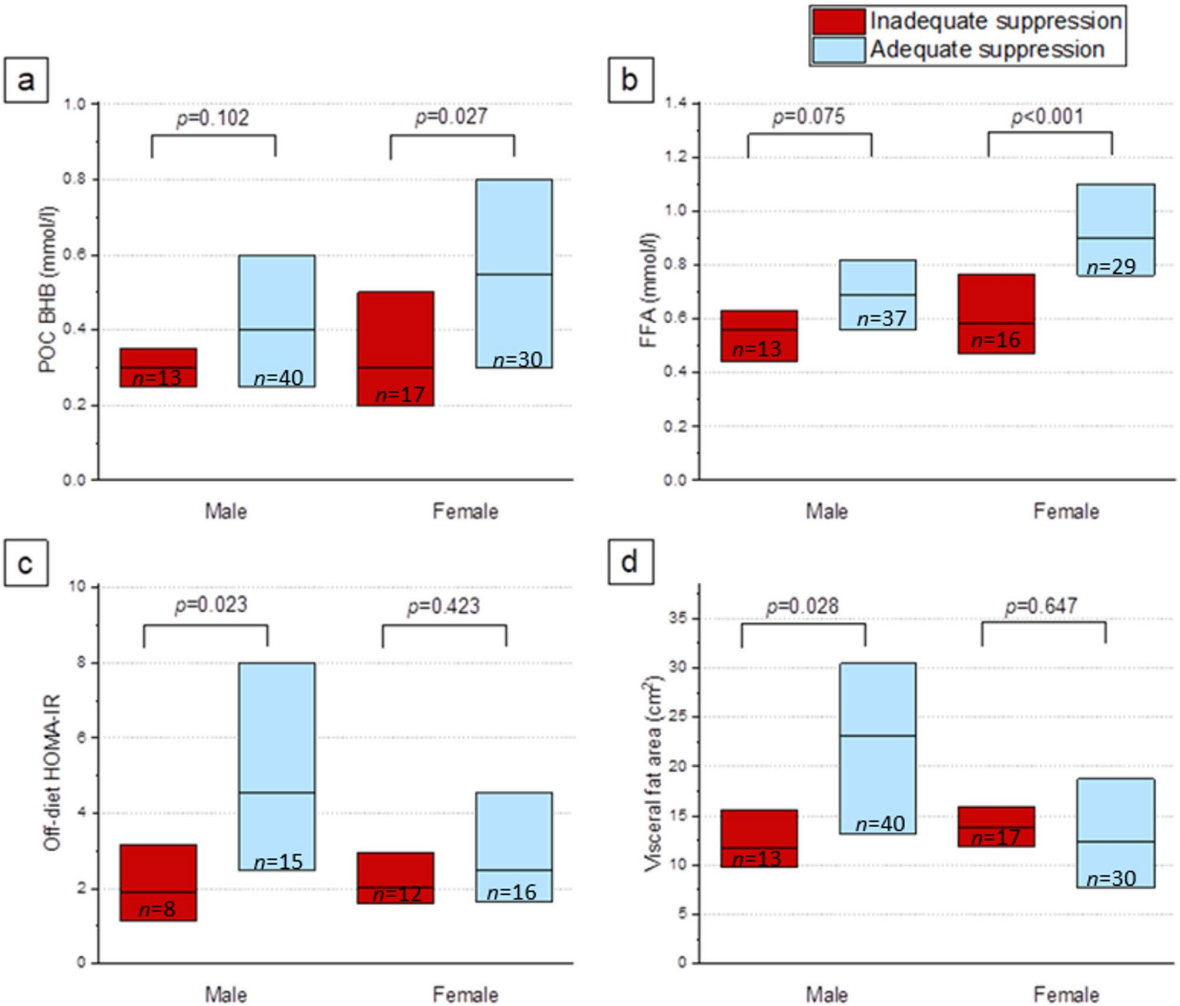
Subgroup analysis in men and women. The boxes extend from the 25th percentile (lower edge) to the 75th percentile (upper edge), with the line inside each box representing the median. In women, adequate suppression was associated with BHB (**a**) and FFA levels (**b**) before [^18^F]FDG injection. In men, adequate suppression was associated with HOMA-IR (**c**) and visceral adiposity (**d**). Abbreviations: POC BHB, point-of-care β-hydroxybutyrate; FFA, free fatty acids; HOMA-IR, homeostatic model assessment– insulin resistance

Overall, 70 patients (70%) showed adequate myocardial suppression. Comparison between those with inadequate and those with adequate suppression can be seen in Table 3. The BHB, FFA and triglyceride levels were lower in the patients with inadequate suppression than in those with adequate suppression. The differences in BHB and FFA were more pronounced in women than in men (Fig. 1). Of the patients being treated with systemic corticosteroids, 14 (88%) had adequate suppression, compared to 56 (67%) without systemic corticosteroids (*p* = 0.096). No differences were noted in the state of suppression in patients taking metformin (*p* = 0.617), SGLT2 inhibitors (*p* = 0.218) or other glucose modulating treatments (*p* = 0.462).

**Table 3:** Clinical characteristics according to the success of sufficient suppression in the entire material and separately for men and women

	Whole study population			Men			Women		
	Inadequate suppression	Adequate suppression	*p*	Inadequate suppression	Adequate suppression	*p*	Inadequate suppression	Adequate suppression	*p*
**On-diet laboratory assays**									
POC BHB (mmol/l)	0.3 (0.1–2.6)	0.4 (0.1–5.8)	**0.011**	0.3 (0.1–0.5)	0.4 (0.1–5.8)	0.102	0.3 (0.1–2.6)	0.6 (0.2-3.0)	**0.027**
BHB (mmol/l)^a^	0.26 (< 0.01–0.60)	0.43 (< 0.01–2.49)	**0.004**	0.27 (< 0.01–0.60)	0.35 (< 0.01–2.11)	0.130	0.25 (< 0.01–0.59)	0.48 (0.12–2.49)	**0.006**
FFA (mmol/l)^b^	0.58 (0.22-1.00)	0.76 (< 0.01–1.44)	**< 0.001**	0.56 (0.33–0.98)	0.69 (0.34–1.40)	0.075	0.59 (0.22-1.00)	0.90 (< 0.01–1.44)	**< 0.001**
Glucose (mmol/l)^c^	5.2 (4.2–7.1)	5.1 (3.7–10.2)	0.376	5.3 (4.6–7.1)	5.1 (3.7–10.2)	0.431	5.1 (4.2–5.8)	4.8 (4.0-7.1)	0.399
Insulin (mU/l)^c^	7.2 (2.8–20.3)	8.3 (1.5–56.6)	0.285	5.8 (3.2–18.0)	8.7 (1.5–56.6)	0.121	8.1 (2.8–20.3)	6.0 (1.5–41.9)	0.764
Total cholesterol (mmol/l)^c^	4.6 (2.8–6.3)	4.1 (2.1–6.8)	0.260	4.6 (3.3–5.8)	4.0 (2.1–6.8)	0.098	4.7 (2.8–6.3)	4.6 (2.1–6.5)	0.936
HDL-C (mmol/l)^c^	1.45 (0.67–2.29)	1.39 (0.47–3.25)	0.292	1.44 (0.93–1.73)	1.14 (0.47–2.18)	**0.047**	1.52 (0.67–2.29)	1.62 (0.67–3.25)	0.475
LDL-C (mmol/l)^c^	3.1 (1.1–4.7)	2.5 (0.4–4.9)	0.093	3.1 (1.7–4.4)	2.5 (0.7–4.9)	0.066	3.2 (1.1–4.7)	2.6 (0.4–4.8)	0.496
Triglyceride (mmol/l)^c^	0.87 (0.44–1.89)	1.12 (0.34–7.33)	**0.028**	0.94 (0.48–1.36)	1.19 (0.49–7.33)	**0.018**	0.83 (0.44–1.89)	0.94 (0.34–3.54)	0.381
HOMA-IR^c^	1.53 (0.55–5.23)	1.81 (0.28–13.22)	0.296	1.31 (0.67–4.80)	2.16 (0.28–12.83)	0.160	1.76 (0.55–5.23)	1.33 (0.28–13.22)	0.747
**Off-diet laboratory assays** ^d^									
BHB (mmol/l)	< 0.01 (< 0.01–0.15)	< 0.01 (< 0.01–0.54)	0.262	< 0.01 (< 0.01–0.12)	< 0.01 (< 0.01–0.46)	0.494	< 0.01 (< 0.01–0.15)	< 0.01 (< 0.01–0.54)	0.820
Glucose (mmol/l)	5.6 (4.6-7.0)	5.6 (4.8–7.7)	0.405	5.7 (5.0–7.0)	5.6 (4.9–7.7)	0.875	5.5 (4.6–5.8)	5.6 (4.8–7.3)	0.260
Insulin (mU/l)	8.5 (2.5–88.8)	13.5 (3.6–48.0)	**0.018**	6.9 (3.6–88.8)	14.6 (5.9–48.0)	**0.028**	9.3 (2.5–21.5)	10.5 (3.6–38.9)	0.478
Total cholesterol (mmol/l)	5.0 (2.9–7.1)	4.4 (2.4–7.4)	0.209	4.7 (3.2–6.3)	4.3 (3.0-7.4)	0.213	5.3 (2.9–7.1)	4.7 (2.4–7.4)	0.537
HDL-C (mmol/l)	1.56 (1.03–3.05)	1.39 (0.63–3.09)	0.289	1.48 (1.04–1.79)	1.21 (0.63–1.63)	0.076	1.73 (1.03–3.05)	1.59 (0.83–3.09)	0.732
LDL-C (mmol/l)	3.3 (1.5–4.8)	2.6 (0.5–5.3)	0.080	3.2 (1.6–4.5)	2.6 (1.6–5.3)	0.169	3.4 (1.5–4.8)	2.6 (0.5–5.2)	0.241
Triglyceride (mmol/l)	1.33 (0.55–3.36)	1.47 (0.50–3.73)	0.380	1.35 (0.55-2.00)	1.38 (0.52–3.73)	0.392	1.32 (0.57–3.36)	1.49 (0.50–3.60)	0.698
HOMA-IR	1.91 (0.63–23.68)	3.72 (0.88–12.62)	**0.016**	1.90 (0.85–23.68)	4.56 (1.28–11.31)	**0.023**	2.05 (0.63–5.54)	2.49 (0.88–12.6)	0.423
**Body composition**									
BMI (kg/m^2^)	29.2 (19.4–40.0)	29.0 (18.4–54.4)	0.886	25.7 (19.4–32.3)	29.4 (20.6–54.4)	0.072	30.9 (20.9–40.0)	28.1 (18.4–47.9)	0.241
Visceral fat area (cm^2^)	13.25 (4.51–30.42)	18.51 (1.56–54.89)	0.162	11.78 (4.51–26.23)	23.08 (4.54–52.11)	**0.022**	13.81 (7.21–30.42)	12.31 (1.56–54.89)	0.388
Subcutaneous fat area (cm^2^)	28.41 (5.74–67.37)	26.39 (5.16–81.98)	0.976	18.36 (5.74–45.94)	24.24 (8.66–59.30)	0.069	36.79 (14.02–67.37)	29.32 (5.16–81.98)	0.388
Liver attenuation (HU)	51.11 (-8.49-63.78)	45.94 (1.14–57.74)	**0.014**	52.60 (38.25–63.78)	43.21 (3.04–56.42)	**< 0.001**	50.58 (-8.49-58.86)	49.48 (1.14–57.74)	0.782
Liver-to-spleen ratio	1.17 (-0.19-1.59)	1.04 (0.03–2.75)	**0.013**	1.16 (0.89–1.36)	1.00 (0.07–2.75)	**0.013**	1.18 (-0.19-1.59)	1.11 (0.03–1.81)	0.376
**Cardiometabolic risk factors**									
Type 2 diabetes	1 (3%)	8 (11%)	0.195	0 (0%)	4 (10%)	0.236	1 (6%)	4 (13%)	0.426
Obesity*	13 (43%)	28 (40%)	0.756	2 (15%)	18 (45%)	0.056	11 (65%)	10 (33%)	**0.038**
Hypertension	6 (20%)	29 (41%)	**0.040**	1 (8%)	19 (48%)	**0.010**	5 (29%)	10 (33%)	0.782
Dyslipidaemia	9 (30%)	36 (51%)	**0.048**	4 (31%)	20 (50%)	0.226	5 (29%)	16 (53%)	0.113
Fatty liver	6 (20%)	30 (43%)	**0.029**	1 (8%)	21 (53%)	**0.004**	5 (29%)	9 (30%)	0.966

Values are presented as medians (range) for continuous variables and as counts (percentages) for discreate variables. *p* < 0.05 is considered significant (bolded). *POC*, a point-of-care device; *BHB*, beta-hydroxybutyrate; *FFA*, free fatty acids; *HDL-C*, high-density lipoprotein cholesterol; *LDL-C*, low-density lipoprotein cholesterol; *HOMA-IR*, homeostatic model assessment– insulin resistance; *BMI*, body mass index; *HU*, Hounsfield units. *Using WHO classification, BMI ≥ 30. a, *n* = 85; b, *n* = 95; c, *n* = 99; d, *n* = 51; all others, *n* = 100

In the off-diet laboratory tests, insulin levels and HOMA-IR were lower in the inadequate suppression group than in the adequate suppression group. However, this difference was significant in men but not in women (Fig. 1). The results excluding patients diagnosed with T2DM were in line with these results (Table 4).

**Table 4:** Clinical characteristics excluding patients with diabetes according to the success of sufficient suppression in the entire material and separately for men and women

	Study population excluding patients with T2DM (n = 91)			Men			Women		
	Inadequate suppression (*n* = 29)	Adequate suppression (*n* = 62)	*p*	Inadequate suppression (*n* = 13)	Adequate suppression (*n* = 36)	*p*	Inadequate suppression (*n* = 16)	Adequate suppression (*n* = 26)	*p*
**On-diet laboratory assays**									
POC BHB (mmol/l)	0.3 (0.1–2.6)	0.4 (0.1–5.8)	**0.025**	0.3 (0.1–0.5)	0.4 (0.1–5.8)	0.110	0.3 (0.1–2.6)	0.6 (0.2-3.0)	0.061
BHB (mmol/l)^a^	0.27 (< 0.01–0.60)	0.41 (< 0.01–2.41)	**0.011**	0.27 (< 0.01–0.60)	0.34 (< 0.01–2.11)	0.161	0.27 (< 0.01–0.59)	0.48 (0.12–2.41)	**0.016**
FFA (mmol/l)^b^	0.58 (0.22-1.00)	0.74 (< 0.01–1.44)	**0.004**	0.56 (0.33–0.98)	0.64 (0.34–1.40)	0.133	0.59 (0.22-1.00)	0.85 (< 0.01–1.44)	**0.002**
Glucose (mmol/l)^c^	5.2 (4.2–7.1)	5.1 (3.7–6.5)	0.240	5.3 (4.6–7.1)	5.1 (3.7–6.5)	0.318	5.0 (4.2–5.5)	4.8 (4.0–6.0)	0.242
Insulin (mU/l)^c^	6.9 (2.8–19.0)	7.3 (1.5–56.6)	0.388	5.8 (3.2–18.0)	8.5 (1.5–56.6)	0.213	7.9 (2.8–19.0)	5.9 (1.5–32.0)	0.738
Total cholesterol (mmol/l)^c^	4.7 (2.8–6.3)	4.1 (2.1–6.8)	0.246	4.6 (3.3–5.8)	4.0 (2.1–6.8)	0.077	4.8 (2.8–6.3)	4.6 (3.0-6.5)	0.883
HDL-C (mmol/l)^c^	1.47 (0.67–2.29)	1.41 (0.47–3.25)	0.548	1.44 (0.93–1.73)	1.21 (0.47–2.18)	0.103	1.54 (0.67–2.29)	1.64 (0.67–3.25)	0.314
LDL-C (mmol/l)^c^	3.2 (1.1–4.7)	2.5 (0.7–4.9)	0.082	3.1 (1.7–4.4)	2.5 (0.7–4.9)	0.070	3.2 (1.1–4.7)	2.6 (1.4–4.8)	0.495
Triglyceride (mmol/l)^c^	0.86 (0.44–1.89)	1.01 (0.34–2.76)	0.072	0.94 (0.48–1.36)	1.18 (0.49–2.76)	**0.044**	0.82 (0.44–1.89)	0.86 (0.34–1.60)	0.583
HOMA-IR^c^	1.50 (0.55–4.80)	1.64 (0.28–12.83)	0.497	1.31 (0.67–4.80)	2.03 (0.28–12.83)	0.308	1.60 (0.55–4.39)	1.25 (0.28–8.11)	0.602
**Off-diet laboratory assays** ^d^									
BHB (mmol/l)	< 0.01 (< 0.01–0.15)	< 0.01 (< 0.01–0.46)	0.551	< 0.01 (< 0.01–0.12)	< 0.01 (< 0.01–0.46)	0.494	< 0.01 (< 0.01–0.15)	< 0.01 (< 0.01–0.11)	1.000
Glucose (mmol/l)	5.6 (4.6-7.0)	5.6 (4.8–7.7)	0.746	5.7 (5.0–7.0)	5.6 (4.9–7.7)	0.697	5.5 (4.6–5.8)	5.4 (4.8–6.3)	0.527
Insulin (mU/l)	8.5 (2.5–88.8)	11.9 (3.6–41.8)	0.054	6.9 (3.6–88.8)	14.6 (5.9–41.8)	**0.037**	9.3 (2.5–21.5)	10.3 (3.6–25.2)	0.860
Total cholesterol (mmol/l)	5.0 (2.9–7.1)	4.4 (3.0-7.4)	0.245	4.7 (3.2–6.3)	4.3 (3.0-7.4)	0.238	5.3 (2.9–7.1)	4.7 (3.4–7.4)	0.595
HDL-C (mmol/l)	1.56 (1.03–3.05)	1.48 (0.89–3.09)	0.591	1.48 (1.04–1.79)	1.21 (0.89–1.63)	0.121	1.73 (1.03–3.05)	1.66 (1.14–3.09)	0.432
LDL-C (mmol/l)	3.3 (1.5–4.8)	2.6 (1.6–5.3)	0.075	3.2 (1.6–4.5)	2.6 (1.6–5.3)	0.161	3.4 (1.5–4.8)	2.6 (1.7–5.2)	0.231
Triglyceride (mmol/l)	1.33 (0.55–3.36)	1.38 (0.50–3.73)	0.644	1.35 (0.55-2.00)	1.38 (0.52–3.73)	0.456	1.32 (0.57–3.36)	1.36 (0.50–2.13)	0.980
HOMA-IR	1.91 (0.63–23.68)	2.83 (0.88–10.59)	0.053	1.90 (0.85–23.68)	4.55 (1.28–10.59)	**0.037**	2.05 (0.63–5.54)	2.37 (0.88–5.71)	0.781
**Body composition**									
BMI (kg/m^2^)	29.1 (19.4–40.0)	28.8 (18.4–54.4)	0.946	25.7 (19.4–32.3)	28.9 (20.6–54.4)	0.147	30.7 (20.9–40.0)	28.1 (18.4–47.9)	0.214
Visceral fat area (cm^2^)	12.69 (4.51–30.42)	18.09 (1.56–52.11)	0.303	11.78 (4.51–26.23)	21.81 (4.54–52.11)	**0.046**	13.25 (7.21–30.42)	10.83 (1.56–28.57)	0.233
Subcutaneous fat area (cm^2^)	27.53 (5.74–67.37)	25.02 (5.16–81.98)	0.851	18.36 (5.74–45.94)	22.74 (8.66–56.80)	0.113	35.51 (14.02–67.37)	26.66 (5.16–81.98)	0.254
Liver attenuation (HU)	51.16 (-8.49-63.78)	46.58 (3.04–57.74)	0.676	52.60 (38.25–63.78)	43.90 (3.04–56.42)	**0.001**	50.82 (-8.49-58.86)	50.01 (25.10-57.74)	0.641
Liver-to-spleen ratio	1.18 (-0.19-1.59)	1.06 (0.07–1.81)	**0.020**	1.16 (0.89–1.36)	1.01 (0.07–1.44)	**0.015**	1.21 (-0.19-1.59)	1.12 (0.55–1.81)	0.468
**Cardiometabolic risk factors**									
Obesity*	12 (41%)	28 (37%)	0.696	2 (15%)	15 (42%)	0.088	11 (65%)	10 (33%)	**0.044**
Hypertension	5 (17%)	25 (40%)	**0.029**	1 (8%)	17 (47%)	**0.011**	5 (29%)	10 (33%)	0.688
Dyslipidaemia	8 (28%)	31 (50%)	**0.044**	4 (31%)	18 (50%)	0.232	5 (29%)	16 (53%)	0.109
Fatty liver	6 (21%)	24 (39%)	**0.029**	1 (8%)	18 (50%)	**0.007**	5 (29%)	9 (30%)	0.559

Values are presented as medians (range) for continuous variables and as counts (percentages) for discreate variables. *p* < 0.05 is considered significant (bolded). *POC*, a point-of-care device; *BHB*, beta-hydroxybutyrate; *FFA*, free fatty acids; *HDL-C*, high-density lipoprotein cholesterol; *LDL-C*, low-density lipoprotein cholesterol; *HOMA-IR*, homeostatic model assessment– insulin resistance; *BMI*, body mass index; *HU*, Hounsfield units. *Using WHO classification, BMI ≥ 30. a, *n* = 80; b, *n* = 86; c, *n* = 90; d, *n* = 40; all others, *n* = 91

Those with inadequate suppression had higher liver attenuation, higher liver-spleen attenuation ratio, and a lower rate of fatty liver than in those with adequate suppression. No differences were detected in visceral or subcutaneous fat areas between the inadequate and adequate suppression groups.

While the male patients were heavier than the females, there was no difference in BMI between the sexes. Among the women, the proportion of individuals with obesity was higher with inadequate suppression compared to women with adequate suppression (65% and 33%, respectively, *p* = 0.038), but this difference was not apparent in men (15% and 45%, respectively, *p* = 0.056). In the male group, the visceral fat area was lower in individuals with inadequate suppression than in those with adequate suppression (Fig. 1). The male patients with inadequate suppression also exhibited a lower prevalence of fatty liver, but this was not observed in female patients.

In the whole population, and especially in men, the prevalence of hypertension was statistically significantly higher in those who achieved adequate myocardial glucose uptake suppression than in those with inadequate suppression.

### Validation of the pretest calculator of adequate suppression

**Fig. 2:**
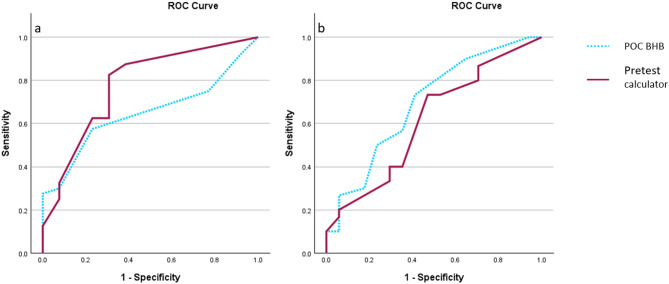
Receiver operating curves of point-of-care measured BHB and pretest calculator to predict adequate suppression, (**a**) in men and (**b**) in women. *POC*, point-of-care; *BHB*, β-hydroxybutyrate

The receiver operating curves can be seen in Fig. 2. In the whole study population, the AUC for the point-of-care BHB measurement to predict adequate suppression is 0.659 (95% confidence interval 0.546–0.771), and for the pretest calculator, the AUC is 0.673 (95% confidence interval 0.553–0.792). The difference was not statistically significant (*p* = 0.860). The AUCs are also similar in women (0.694 for BHB, 0.614 for pretest calculator, *p* = 0.483). In men, the curves are visually different, but according to the DeLong test, the difference in AUC is not statistically significant (0.649 for BHB, 0.778 for the pretest calculator, *p* = 0.226), nor is it significant between men and women for either the calculator (*p* = 0.126) or the BHB test (*p* = 0.923).

Using cut-off value of 0.35 mmol/l for the point-of-care BHB and 15.5% probability of inadequate suppression for the calculator, both tests showed similar sensitivity and specificity (64% and 67% for the BHB, 67% and 63% for the pretest calculator). Both tests have higher specificity in men (77% for the BHB and 77% for the pretest calculator) than in women (59% for the BHB and 53% for the pretest calculator).

In patients with low BHB level, there were no differences in laboratory findings or body composition between those with inadequate and those with adequate suppression. However, in men with low BHB levels, the off-diet HDL-C levels were higher in patients with inadequate suppression (median 1.48 (1.04–1.79) mmol/l) than those with adequate suppression (1.16 (0.63–1.50) mmol/l, *p* = 0.043). The rates of obesity (10%) and hypertension (10%) were also lower in male patients with inadequate suppression than in those with adequate suppression (53% and 47%, respectively, *p* = 0.026 and *p* = 0.049, respectively).

In all patients with a higher than 15.5% probability of inadequate suppression, the rate of dyslipidaemia was lower in patients with inadequate suppression than in those with adequate suppression. In men, there was a lower rate of obesity in the inadequate suppression group than in the adequate suppression group (10% and 53%, respectively, *p* = 0.027). The differences between inadequate and adequate suppression groups did not reach statistical significance for the rates of hypertension (10% and 47%, respectively, *p* = 0.054) and dyslipidaemia (30% and 67%, respectively, *p* = 0.072). In women with low BHB or with a higher than 15.5% probability of inadequate suppression, there were no differences between the suppression groups.

## Discussion

False positive findings related to inadequate myocardial [^18^F]FDG uptake suppression are a common problem in cardiac PET-CT. Elevated BHB detected in pre-imaging measurements has been proposed to be a sign of successful fasting and dieting and, thus, a predictor of adequate myocardial glucose uptake suppression. However, some patients have adequate suppression even with low BHB levels, likely reflecting reduced uptake due to myocardial insulin resistance, rather than successful intervention through diet and fasting. Our study aimed to evaluate whether reduced myocardial [^18^F]FDG uptake beyond what is expected during a short-term ketogenic diet reflects the presence of an underlying cardiometabolic disorder. We found that cardiometabolic risk factors, such as insulin resistance, hypertension, dyslipidaemia, and visceral adiposity, were significantly associated with reduced myocardial glucose uptake. However, this association appeared to be sex-dependent.

**Fig. 3:**
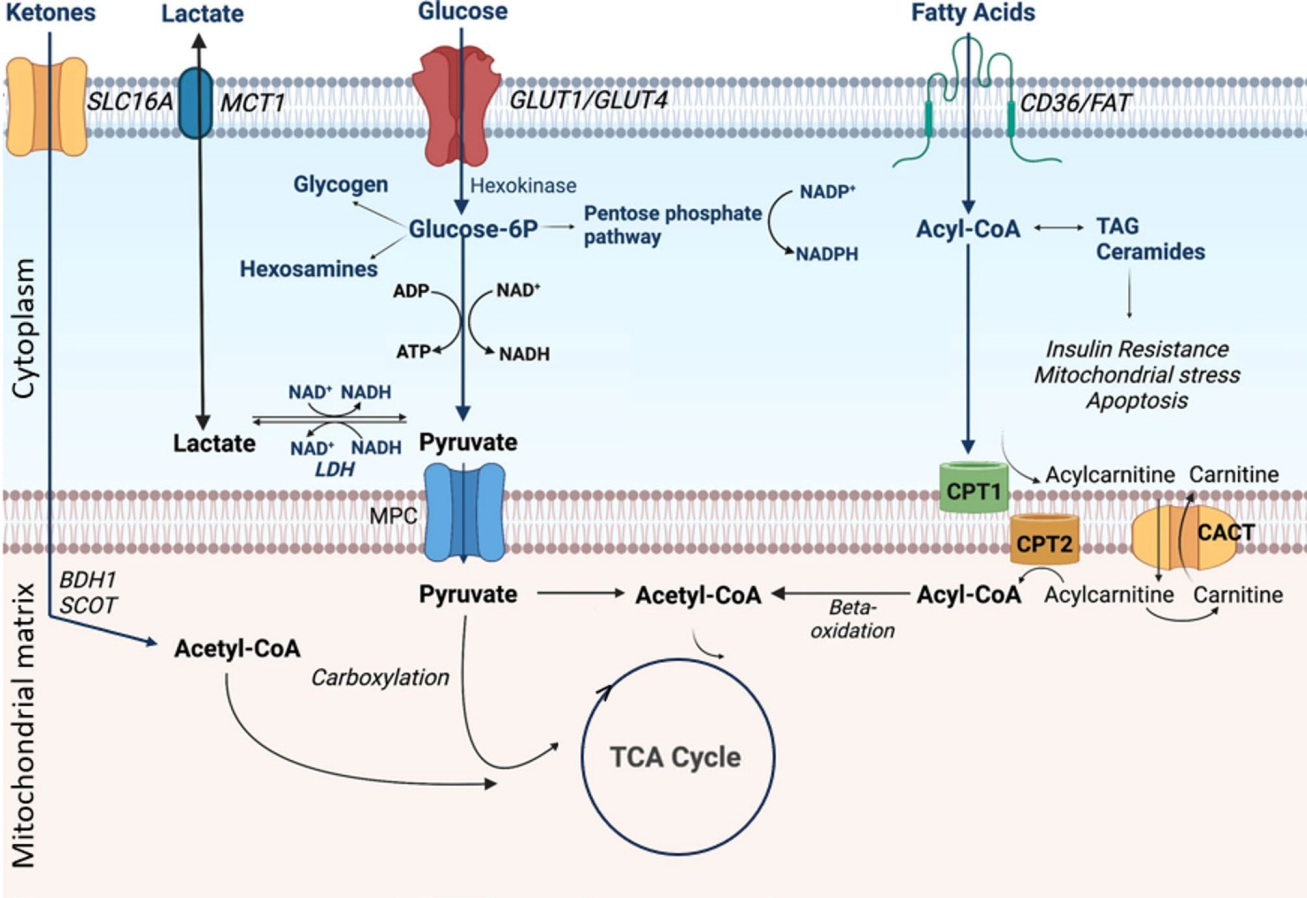
Overview of metabolic pathways in normal heart Modified from Ng et al., 2023 [27]. CC-BY 4.0

An overview of myocardial metabolism is shown in Fig. 3. After a meal, glucose serves as the primary energy source for the myocardium. Uptake of glucose in adults is facilitated by glucose transporter type 4 (GLUT4). The translocation of GLUT4 to the sarcolemma is stimulated by insulin [23]. During fasting conditions, insulin levels are reduced, leading to reduced glucose uptake and promoting a shift towards the use of FFA, which can diffuse through the sarcolemma freely or via fatty acid translocase (FAT) [24]. During prolonged fasting or carbohydrate restriction, ketone bodies, primarily BHB, become important alternative energy substrates [25]. In T2DM, insulin signalling in adipocytes is impaired, leading to inadequately suppressed lipolysis and increased blood FFA levels, even with relatively low ketone levels. FFA uptake is also increased, as both insulin resistance and relative insulin deficiency reduce GLUT4 translocation to the sarcolemma [26]. Prolonged alterations in FFA metabolism can have broader health consequences, including the induction of metabolic syndrome.

Metabolic syndrome is a combination of risk factors associated with atherosclerosis and T2DM. Multiple sets of criteria have been published through the years, all highlighting (central) obesity, insulin resistance, dyslipidaemia and high blood pressure [18–20]. Many imaging modalities can hint at metabolic syndrome traits, including visceral adiposity [21]. Succurro et al. reported in 2022 that metabolic syndrome is associated with attenuated myocardial glucose uptake in patients with T2DM [22]. However, the sample size was limited and restricted to patients with diabetes. Later, Devesa et al. conducted a comprehensive study showing that having multiple metabolic syndrome traits is associated with myocardial insulin resistance [8].

Many individual risk factors for cardiometabolic syndrome have been associated with altered myocardial glucose metabolism in non-ketogenic conditions. Kim et al. published in 2015 that visceral adiposity is an independent risk factor for attenuated [^18^F]FDG uptake after eight hours of fasting [7]. This is in line with our findings. Moreover, fatty liver in its different stages is associated with altered [^18^F]FDG uptake [4, 5, 28]. In the present study and as previously reported, this association is strong enough to be seen in the setting of the ketogenic diet. In the present study, the off-diet HOMA-IR value showed an inverse correlation with myocardial uptake. This has been shown before in non-ketogenic settings [29]. Hypertension, on the other hand, is a hallmark of cardiometabolic syndrome [30], as is supported by our results showing a strong association with reduced uptake in men, but no association in women.

A study by Devesa et al., used a population consisting mainly of men, though the authors adjusted the results by age and sex [8]. In a recent study by Succurro et al., type 2 diabetes, as well as prediabetes, was associated with more significant impairment of myocardial glucose uptake in women than in men [31]. In our study, traits associated with metabolic syndrome were linked to reduced myocardial uptake, particularly in men. This reduction was evident even under fasting conditions following a ketogenic diet.

The male heart has been shown to be less prone to utilize glucose than the female heart [32]. However, oestrogen has been suggested to upregulate nitric oxide synthases and thus reduce glucose transporter (GLUT) type 4 translocation to the cellular membrane [33]. The same study showed higher glucose metabolism relative to insulin level in the female heart than in the male heart. However, in the present study, the female population was likely largely postmenopausal, judging by their age, and therefore lacking in oetrogen.

Interestingly, our results show that while the BMI did not differ between men and women, the myocardial [^18^F]FDG uptake was higher in women with obesity than in women who were not obese. This may reflect the fact that women, particularly premenopausal women, tend to remain insulin-sensitive despite increased subcutaneous fat accumulation, a phenomenon less commonly observed in men. Moreover, myocardial suppression was associated with visceral adiposity, liver attenuation and fatty liver only in men, not in women. In the literature, low skeletal muscle mass has been associated with reduced myocardial glucose uptake in men, highlighting both the body composition and sex-dependence of insulin resistance-related myocardial substrate metabolism [34].

Our findings have some clinical implications. First, the results suggest that male patients with cardiometabolic disorders tend to have reduced myocardial glucose uptake and thus have a higher probability of adequate suppression in diagnostic [^18^F]FDG PET/CT of inflammatory cardiac diseases. Second, cardiometabolic disorders could affect viability studies, although ischaemia induces GLUT1 overexpression, making insulin resistance in the ischaemic region unlikely to be problematic [35].

A portion of our study population was on systemic corticosteroid treatment. While the association between corticosteroids and adequate suppression was not statistically significant, this may be due to type II error, (i.e. the small study population).

Our study has some limitations. First, we gave heparin to patients with low BHB measured with a point-of-care device. Heparin increases the possibility of adequate suppression following a ketogenic diet and elongated fasting [11, 12, 36]. However, as we have shown before, heparin does not significantly affect suppression in patients with low BHB level [37]. Second, we had a high drop-out rate regarding laboratory testing on a separate extra visit. We measured insulin resistance off-diet assuming that myocardial insulin resistance would be associated with the long-term overall insulin resistance measured by HOMA-IR. The short-term ketogenic diet might lower the need for insulin in relation to glucose because there is a high amount of competitive nutrients available, and thus might not reflect actual insulin resistance. Although the drop-out rate was similar in men and women, it was higher in patients with adequate suppression and does reduce the study’s power to show some of the metabolic connections, especially in the subgroups. Thirdly, we did not measure FFA levels off-diet to show reliance on FFA metabolism in patients with insulin resistance, nor did we measure haemoglobin A1c to rule out undiagnosed T2DM or prediabetes. We suggest that these should be investigated in future studies. Furthermore, our study population was heterogenous in terms of BMI. Although this reflects our clinical population, the phenomenon could be studied in a population with less variation in BMI in the future. Additionally, our study was performed under a short-term ketogenic diet. Thus, the results cannot be extrapolated to other [^18^F]FDG imaging protocols.

## Conclusions

We conclude that even during short-term ketogenic diet, cardiometabolic disease are associated with reduced myocardial glucose uptake especially in men, likely due to impaired insulin signalling. However, this may have advantages in diagnostic cardiac PET/CT imaging when the purpose is to distinguish inflammatory changes from the normal myocardial metabolic activity. In women, factors other than FFA and BHB do not markedly predict the suppression of myocardial glucose uptake.

## Data Availability

The data underlying this article cannot be shared publicly due to the privacy of individuals that participated in the study. The statistical analysis output data will be shared on request to the corresponding author.

## Abbreviations

AUC: Area under ROC curve
BHB: β-hydroxybutyrate
BMI: Body mass index
CT: Computed tomography
FDG: Fluorodeoxyglucose
GLUT: Glucose transporter
HDL-C: High-density lipoprotein cholesterol
HOMA-IR: Homeostatic model assessment– insulin resistance
HU: Hounsfield unit
LDL-C: Low-density lipoprotein cholesterol
PET: Positron emission tomography
POC: Point-of-care
ROC: Receiver operating curve
ROI: Region of interest
SGLT2: Sodium-glucose cotransporter 2
SUV_LV_: Mean of maximum standardised uptake values of left ventricle
SUV_max_: Maximum standardised uptake value
SUVratio: SUV_LV_ to left ventricle blood pool ratio
T2DM: Type 2 diabetes mellitus
